# Closing the loop: from paper to protein annotation using supervised Gene Ontology classification

**DOI:** 10.1093/database/bau088

**Published:** 2014-09-04

**Authors:** Julien Gobeill, Emilie Pasche, Dina Vishnyakova, Patrick Ruch

**Affiliations:** ^1^BiTeM group, University of Applied Sciences—HEG, Library and Information Sciences, Rte de Drize 7, 1227 Geneva, Switzerland, ^2^Division of Medical Information Sciences, University and Hospitals of Geneva, Geneva, Switzerland and ^3^SIBtex group, SIB Swiss Institute of Bioinformatics, Rue Michel-Servet 1, 1206 Geneva, Switzerland

## Abstract

Gene function curation of the literature with Gene Ontology (GO) concepts is one particularly time-consuming task in genomics, and the help from bioinformatics is highly requested to keep up with the flow of publications. In 2004, the first BioCreative challenge already designed a task of automatic GO concepts assignment from a full text. At this time, results were judged far from reaching the performances required by real curation workflows. In particular, supervised approaches produced the most disappointing results because of lack of training data. Ten years later, the available curation data have massively grown. In 2013, the BioCreative IV GO task revisited the automatic GO assignment task. For this issue, we investigated the power of our supervised classifier, GOCat. GOCat computes similarities between an input text and already curated instances contained in a knowledge base to infer GO concepts. The subtask A consisted in selecting GO evidence sentences for a relevant gene in a full text. For this, we designed a state-of-the-art supervised statistical approach, using a naïve Bayes classifier and the official training set, and obtained fair results. The subtask B consisted in predicting GO concepts from the previous output. For this, we applied GOCat and reached leading results, up to 65% for hierarchical recall in the top 20 outputted concepts. Contrary to previous competitions, machine learning has this time outperformed standard dictionary-based approaches. Thanks to BioCreative IV, we were able to design a complete workflow for curation: given a gene name and a full text, this system is able to select evidence sentences for curation and to deliver highly relevant GO concepts. Contrary to previous competitions, machine learning this time outperformed dictionary-based systems. Observed performances are sufficient for being used in a real semiautomatic curation workflow. GOCat is available at http://eagl.unige.ch/GOCat/.

**Database URL:** http://eagl.unige.ch/GOCat4FT/

## Introduction

The problem of data deluge in proteomics is well known: the available curated data lag behind current biological knowledge contained in the literature (1–3), and professional curators need assistance from text mining to keep up with the flow of published discoveries (4–6). One particularly time-consuming and labor-intensive task is gene function curation of articles with Gene Ontology (GO) concepts. Such curation from literature is a highly complex task because it needs expertise in genomics, and also in the ontology itself. For that matter, this task was studied since the first BioCreative challenge in 2005 (7) and is still considered as both unachieved, and long awaited by the community (8).

Our group already participated in this first BioCreative. At this time, we extracted GO concepts from full texts with EAGL, a locally developed dictionary-based classifier (9). EAGL achieved competitive performances among other systems during this BioCreative challenge, or in further independent studies against MetaMap (10). Dictionary-based approaches tend to exploit lexical similarities between the information about GO concepts (descriptions and synonyms) and the input text. They constituted the most evaluated approaches at this time (11, 12), and they are continued to be investigated (13, 14); today, they are integrated in ontology-based search engines such as GoPubMed (15) or in real curation workflows such as Textpresso (16). Yet, dictionary-based approaches are limited by the complex nature of the GO: identifying GO concepts in text is highly challenging, as they often do not appear literally or approximately in text (e.g. for the concept GO:0045196 ‘establishment or maintenance of neuroblast polarity’). Another smaller part of systems evaluated in BioCreative I relied on machine learning approaches. Such algorithms empirically learn behaviors from a knowledge base (KB) that contains training instances, i.e. instances of already curated articles. At that time, machine learning approaches produced the lowest results; the lack of a standard training set was notably pointed out (7).

We recently reported on GOCat (17, 18), our new machine learning GO classifier. GOCat exploits similarities between an input text and already curated instances contained in a KB to infer a functional profile. GO annotations (GOA) and MEDLINE now make it possible to exploit a growing amount of almost 100 000 curated abstracts for populating this knowledge base. Evaluated on the first BioCreative benchmark, GOCat achieved performances close to human curators, with 0.65 for recall at 20 (i.e. 65% of the expected GO concepts present in the first 20 concepts returned by the system), against 0.26 for our dictionary-based classifier. Moreover, we showed in (18) that the quality of the GO concepts predicted by GOCat continues to improve across the time, thanks to the growing number of high-quality GO concepts assignments available in GOA: since 2006, GOCat performances for predicting GO concepts from a just published abstract have improved by 50%.

**Figure 1.**
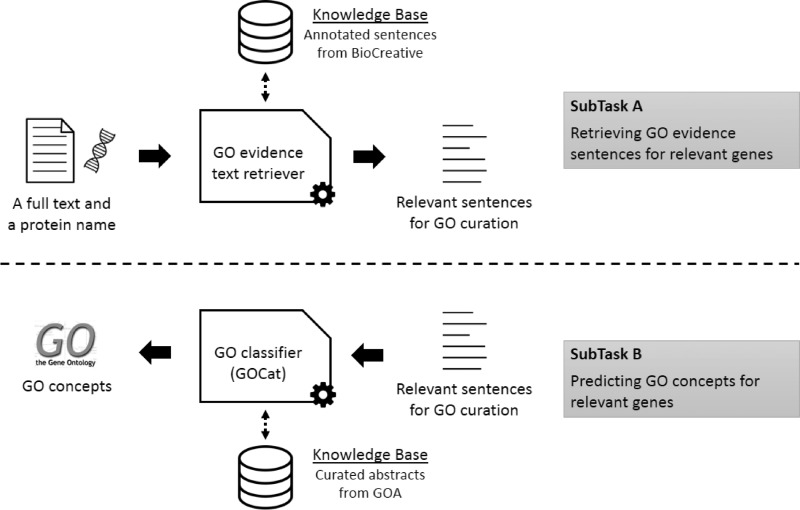
Overall workflow of the BiTeM/SIBtex system for BioCreative IV GO task. First (subtask **A**), given a full text and a protein name, the system extracts relevant sentences for GO curation. Then (subtask **B**), given these relevant sentences, the system predicts relevant GO concepts for curation. For both subtasks, the system uses machine learning, thanks to KB designed from the BioCreative training data and GOA.

The BioCreative IV GO task was the occasion to investigate the GOCat power in a reference challenge. The subtask A aimed at evaluating systems for filtering relevant sentences for GO curation, given a gene name (along with a NCBI gene ID) and a full text (along with is PMID). For this subtask, we designed a GO evidence text retriever, based on a robust state-of-the-art approach, using a naïve Bayes classifier and the official training set. The official training set was provided by the organizers, and was the result of the comprehensive analysis of 100 full texts by a team of collaborating curators (19). It was composed of blocks of sentences that were annotated as relevant for curation, or non-relevant. In machine learning, such examples are called positive or negative instances, respectively. The official training set finally contained 1346 positive instances. Another set of 50 full texts were annotated by the collaborating curators to obtain a development set. The development set was provided for tuning issues. Finally, a final set of 50 full texts was annotated for building the test set. The test set is composed of unseen full texts provided without annotations and used for the competition. On the other hand, we also investigated exploiting GeneRIFs (http://www.ncbi.nlm.nih.gov/gene/about-generif) for an alternative bigger training set (76 000 positive instances). Then, the goal of the subtask B was to use the previously predicted relevant sentences for assigning GO concepts to the given gene. For this subtask, the GO classifier we used was GOCat. In this BioCreative challenge, participants were allowed to submit up to three runs. We thus submitted results computed with GOCat with different numbers of proposed GO concepts: 5, 10 or 20. Figure 1 illustrates the overall workflow of the complete task.

## Material and methods

### Subtask A: retrieving GO evidence sentences for relevant genes

The goal of the subtask A was to determine, given a training set of curated sentences, whether new sentences are relevant for curation or not, and, if possible, to support the decision with a confidence score. Some state-of-the-art methods suitable for such supervised binary classification task include naïve Bayes classifiers and support vector machines (20, 21). For implementation reasons, we chose a naïve Bayes.

As we mentioned above with the GOCat description, we are used to working with statistical GO classification at the abstract/paragraph level, but we rarely apply our system at the sentence level. Thus, for this subtask A, we first further analyzed the data to design a training set, and finally made some strong assumptions about them. We studied the length of evidence texts: as mentioned in the BioCreative guidelines (19), the evidence texts for GOA may be derived from a single sentence, or multiple continuous, or discontinuous, sentences. In the training data, 66% of evidence texts contained only one sentence, 20% contained two sentences and 14% three and more. Hence, our first assumption was to consider only sentences: for example, a block of three positive sentences was considered as three independent positive sentences. Then, we compared, given a full text and a gene name, the set of the positive sentences and the set of sentences where we were able to identify the gene name. In BioCreative IV, the gene names were provided along with a NCBI gene ID. For retrieving a given gene name in sentences, we relied on pattern matching. With a simple case-insensitive mapping, we found the given gene name in 65% of the positive sentences. Then, we searched hyphens in gene names and generated a couple of variants (e.g. for ‘rft-1’ we also tried to map ‘rft1’). With this rule, we reached 80% of sentences detected. We then investigated how to exploit the gene ID and find synonyms and variants in reference databases, but we quickly concluded that this strategy would have brought too much noise than recall. A further look to the data revealed that for most sentences in the 20% missed, the gene name was not explicit but often mentioned via pronouns, or such grammatical expressions that require a syntactic analysis and that is beyond statistical approaches. Hence, we accepted this limit, and our second assumption was to consider only the sentences that contained the gene name. So, 80% of the positive sentences contain the gene name. On the other hand, 20% of the sentences that contain a given gene name are positive sentences (i.e. relevant for GO curation) and 80% are negative. This was our third assumption: the training data should contain this 4:1 ratio, four negative sentences for one positive sentence. Finally, for the design of training data, we replaced all the gene names we identified by the word ‘genemention’.

We thus were able to design training sets for our naïve Bayes classifier. For our first official run, we built the training set from the official training set that contained 100 curated articles. With our assumptions, we finally obtained a set of 9251 sentences containing gene names: 1346 positives and 7905 negatives. The ratio is slightly different (85% of negatives), possibly because positive sentences can apply for several enumerated genes. For our second run, we added the development set (50 curated articles) to the previous training set, and thus obtained 683 supplementary positive sentences and 3912 supplementary negative sentences.

Finally, we investigated a most ambitious way for designing our training set, based on GeneRIFs. GeneRIFs are concise phrases identified in journal papers and describing a protein function, recorded in the reference databases by PubMed users, which are not always curators. GeneRIFs are not GOA, but potentially provide positive sentences for our task. We first downloaded all available GeneRIFs. In July 2013, there were ∼826 000 entries in the database. Each entry is provided with the gene ID, the GeneRIF text and the PMID that was used. As GeneRIFs are taken in full texts, we considered only those papers whose full text was available in PubMed Central (PMC; http://www.ncbi.nlm.nih.gov/pmc/). For this purpose, we accessed the open access subset of PMC on September 2013, via the dedicated File Transfer Protocol (FTP) services. We then automatically scanned the papers to find sentences that were registered as GeneRIFs. We were able to locate 76 000 GeneRIFs in 48 000 full texts. Thus, these 76 000 GeneRIFs were considered as positive sentences. For negative sentences, we first retrieved all sentences containing the given gene names in the same papers, and considered that all non-positive sentences were negative, which we knew was a too strong assumption. We finally sampled this negative set to keep the 4:1 ratio between positive and negative instances. As for the first training sets, we replaced all identified gene names by ‘genemention’. This GeneRIFs training set was used for producing our third and last run.

Hence, these three training sets were used to train a naïve Bayes classifier. Each word of the collection was considered as a feature. We also add several meta- features, such as the type of section (paragraph, title, caption, etc.), the relative position of the sentence in the full-text (an integer between 1 and 20), the percentage of common words with the abstract and the sentence length. Once the classifier was trained, we parsed the test set. For each article and each gene, we extracted the sentences that contained the gene name. Then, each sentence was sent to the classifier and obtained a class (positive or negative) and a confidence score. As only 20% of sentences that contained a given gene name were positive in the training set, we chose to select only the first 20% best-ranked sentences.

### Subtask B: predicting GO terms for relevant genes

The goal of the subtask B was to predict GO concepts for a given gene in a full text. For this purpose, we used our GO classifier GOCat. GOCat relies on a *k*-nearest neighbors (*k*-NN), a remarkably simple algorithm that assigns to a new text the categories that are the most prevalent among the *k* most similar instances contained in the KB (22). The GOCat KB contains nearly 100 000 MEDLINE abstracts that were used for manual GO curation in the GOA database. Concretely, the GOA annotations that are linked to a PMID are collected in the official GOA Web site: this represents an amount of almost 300 000 (PMID; GO ID) couples. Then, for all PMIDs involved in these annotations, abstracts are collected via the National Library of Medicine e-utils: this represents an amount of almost 100 000 abstracts. Then, all abstracts are indexed in a search engine. For this purpose, we used the Terrier platform (23). GOCat is comprehensively described in (18). For these experiments, the GOA release used for deriving the GOCat KB was downloaded on August 2013.

We discarded all the PMIDs contained in the test set from the knowledge base. Predicting GO concepts for PMIDs that already were in the KB would have caused a bias. Then, we started from the output of our first run. For each article and each gene name, we built a paragraph with the selected sentences, and then we sent the paragraph to GOCat. GOCat was used with *k* = 100. As the *k*-NN usually outputs all possible GO concepts along with a confidence score, we kept only the five most confident GO concepts for our most precision-oriented run, the 10 most confident for a balanced run and the 20 most confident for our most recall-oriented.

### BioCreative submissions formats and metrics

For each subtask, participants were allowed to submit up to three runs. Runs are files that contain system’s output for the whole test set. For the subtask A, given a paper ID and a gene ID, the system had to output sentences that were relevant for GO curation. An example of output line is ‘10995441 32703 13349 298’; the first number is a PMID, the second is a NCBI gene ID and the last two are offsets (position and length) of the returned sentence. For the subtask B, the system had to output lines that contained predicted GO concepts, given a paper ID and a gene ID. An example of output line is ‘10995441 32703 GO:0005515, where the first number is a PMID, the second is a gene ID and the third field is the predicted GO ID. Participants’ runs were compared with the so-called gold standard. The gold standard is the curation made by the collaborative curators. Concretely, the gold standard is a file that contains all the correct associations to predict.

For evaluation, traditional metrics were used, such as precision, recall and F measure (F1). Precision is the portion of returned associations that were correct (in the gold standard). Recall is the portion of expected associations (the gold standard) that were returned by the system. Finally, F1 is the harmonic mean of recall and precision. For the subtask A, runs were evaluated with a strict and a partial match metrics: the partial match was more relaxed, as a returned text snippet was considered as correct if it included an expected one. In the same way, for the subtask B, a standard metric was computed, with strict evaluation: only the expected GO concepts were considered as correct. But a more relaxed metric was also computed: the hierarchical metric exploited the hierarchical nature of the GO for taking into account predictions that were close to the expected GO concept. For this metric, a predicted association was not correct (=1) or false (=0), but was the portion of common ancestors in both the computer-predicted and human-annotated GO concepts (24).

## Results

### Subtask A

**Figure 2.**
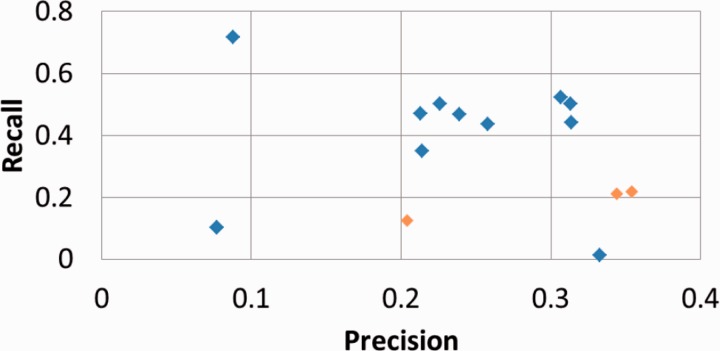
Official results of all competing systems for BioCreative IV subtask A, with partial match metrics. BiTeM/SIBtex results are in orange.

Table 1 presents our results for the subtask A, computed with the official evaluation script and the partial match metrics (19). Figure 2 plots our results within the performances of all competing systems.

**Table 1.** Official results of BiTeM SIBtex for BioCreative IV subtask A with partial match metrics

Precision	Recall	F1	Training set for Naive Bayes
0.344	0.213	0.263	Official training set
**0.354**	**0.22**	**0.271**	Official training and development set
0.204	0.127	0.156	GeneRIFs training set

Given a paper and a gene name, the systems had to propose sentences that were meant to be relevant for GO curation. Precision is the portion of proposed sentences that were correct, recall is the portion of expected sentences that were proposed and F1 is the harmonic mean. The first two runs were obtained with the official training data, and the third was obtained with the GeneRIFs training set designed by our group for this task. Best results for each metric are in bold.

We observe from the table that the best results were obtained by the first two runs, computed with the official training and development set. The contribution of the development set in regards to performances is manifest but light: +3% for F1. The third run was significantly weaker ∼−50% for F1), while the used training set was 40 times bigger. For all runs, we notice that the reached precision was significantly higher than recall. However, the figure confirms that a relatively high precision is the strength of our system, as it produced two leading runs for precision, while it was in the background for reaching high recall compared with the other competing systems.

### Subtask B

**Figure 3.**
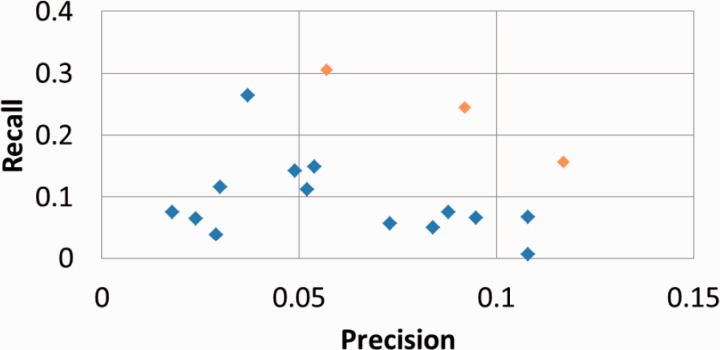
Official results of all competing results for BioCreative IV subtask B with strict metrics. BiTeM/SIBtex results are in orange.

Table 2 presents our results for the subtask B, computed with the official evaluation script, with standard or hierarchical metrics (14). Figure 3 plots our results within the performances of all competing systems.

**Table 2.** Results for the subtask B, computed with the official evaluation script, with standard or hierarchical metrics (14)

Metrics	Precision	Recall	F1	Number of GO concepts returned
Standard	0.117	0.157	0.134	5
Hierarchical	**0.323**	0.356	**0.339**	
Standard	0.092	0.245	0.134	10
Hierarchical	0.248	0.513	0.334	
Standard	0.057	0.306	0.096	20
Hierarchical	0.179	**0.647**	0.280

As expected, the precision is the highest for five concepts returned, and the recall is the highest for 20 concepts returned. The best F1 is observed with 5 and 10 concepts returned. For all runs, the reached recall is significantly higher than the precision. However, the figure shows that GOCat outperformed the other competing systems in the whole range of the evaluated properties.

We can compare these GOCat performances with the performances we observed in previous studies. In (18), GOCat was evaluated on its ability to retrieve GO concepts that were associated with a given PMID, without taking account of the gene. For recall at rank 20 (R20), GOCat achieved performances ranging from 0.56 for new published articles to 0.65 for BioCreative I test set with standard metrics. These performances were obtained by using the abstract for the input text. In this subtask B, the observed standard R20 is 0.306. But this performance was obtained by taking account of the gene, as the input was a set of sentences dealing with a given gene, and the output was GO concepts relevant for this gene. Anyway, these performances are beyond the maximum performances observed in (18) with dictionary-based approaches, which exploit similarities between the input text and GO concepts themselves.

## Discussion

The BioCreative GO task has provided high-quality training data for machine learning, thanks to collaborative curators that screened 100 full texts to have comprehensive sets of relevant (positive) and non-relevant (negative) evidence sentences for GO curation for given genes. Machine learning approaches exploit positive instances to learn to recognize relevant sentences, and exploit negative sentences to learn to recognize irrelevant sentences. These data show that 80% of the relevant evidence sentences contained the gene name as itself (or with a simple hyphen variation). Moreover, on all sentences that contained the gene name, 20% were relevant for curation. We exploited these facts to design a robust and powerful strategy for detecting evidence sentences in an unseen full text.

For the subtask A, our runs were computed with a state-of-the-art statistical approach. We relied on simple and strong assumptions for building a training set from the official data, and used a simple naïve Bayes classifier for filtering sentences. When looking at the others participants’ systems, our performance is fair, as our system produced the leading runs for precision. Nevertheless, our most ambitious strategy was to exploit the GeneRIFs and PubMed Central for building a 40 times more massive training set, but this strategy produced disappointing results. There obviously was a quality problem in the GeneRIFs training set. First, its positive instances were built on the assumption that GeneRIFs are relevant sentences for GO annotation. This assumption seems a priori true, but maybe curators would make some distinctions between these two roles. In particular, some of the GeneRIFs focus on diseases, which are not in the scope of the GO. Moreover, the quality of these sentences for supporting a GO curation is questionable, as they were provided by PubMed users and not edited by NCBI staff. But the weakest point seems to be the building of the negative set, i.e. sentences that are used for learning to recognize irrelevant sentences. For the GeneRIFs training set, we considered that all sentences in the full text that mentioned the gene and were not positive were negative. Yet, GeneRIFs do not aim to produce an exhaustive set of evidence sentences in a full text, but keep only one sentence as evidence, while the annotation was exhaustive in the official BioCreative training set. This means that, in a paper, if several sentences were relevant for the GO curation of one concept, only one was kept for the GeneRIF. Thus, there were 13 positive sentences per article in the BioCreative training set, against 1.6 in our GeneRIFs training set. The probability of false-negative sentences (i.e. sentences that are considered as not relevant while they are) in the GeneRIFs training set thus is high and could mainly explain this counter-performance.

The subtask B was charted territory; we just exploited the power of our supervised classifier GOCat for producing the leading results. Thanks to its KB designed from curated articles in GOA, GOCat is able to propose GO concepts that do not appear literally or even approximately in text. For instance, for the PMID 23840682, GOCat retrieved most of the exact curation, including not only simple concepts such as ‘chloroplast’ or ‘plastid’, but also high-level concepts such as ‘chlorophyll biosynthetic process’ or ‘thylakoid membrane organization’ that are impossible to retrieve for dictionary-based systems. Recall is the main asset of GOCat; for this subtask, the evaluated recall at rank 20 (R20) reached 0.306. Regarding hierarchical metrics, it is surprising to observe such a difference (R20 0.647), while GOCat aims at returning the GO concepts that were most used by curators in GOA. Yet, this performance is remarkable, and is promising in a workflow where the curators would give the gene name and the PMID, then screen and check the proposed GO concepts. In a fully automatic workflow, the best setting would be to return five GO concepts. In this case, the observed F1 (0.134) still is far from human standards for strict curation, but the hierarchical F1 (0.339) seems sufficient for producing added value data. In this perspective, GOCat was used to profile PubChem bioassays (25), or within the COMputational BRidges to EXperiments (COMBREX) project to normalize functions described in free text format (26).

The main limit of GOCat, both observed by reviewers and mentioned in our papers, was the difficulty to integrate it in a curation workflow: it is stated that GOCat proposes more accurate GO concepts, but these concepts are inferred from the whole abstract, then the curators still have to locate the function in the publication and to link the correct GO concept with a gene product. Thanks to BioCreative IV, we were able to design a complete workflow for curation and to evaluate it. Observed performances are sufficient for being used in a real semiautomatic curation workflow. GOCat is available at http://eagl.unige.ch/GOCat/, and the complete pipeline for full-text described in this article is available at http://eagl.unige.ch/GOCat4FT/. In GOCat4FT, the user has to input a gene name and a full text (or a PMC identifier), and the system will display evidence sentences for GO curation (subtask A), and GO concepts provided by GOCat (subtask B).

## Funding

Swiss National Fund for Scientific Research [BiND project 3252B0-105755] and by the FP7 program [Khresmoi project FP7–257528]. Funding for open access charge: University of Applied Sciences Western Switzerland, Geneva (HEG/BiTeM/HES-SO Geneve).

*Conflict of interest*. None declared.

## References

[1] Blake J.A. Bult C.J. (2006) Beyond the data deluge: data integration and bio-ontologies. J. Biomed. Inform., 39, 314–320 16564748 10.1016/j.jbi.2006.01.003

[2] Howe D. Costanzo M. Fey P.*.* (2008) Big data: the future of biocuration. Nature, 455, 47–50 18769432 10.1038/455047a PMC2819144

[3] Baumgartner W. Cohen K.B. Fox L.*.* (2007) Manual curation is not sufficient for annotation of genomic databases. Bioinformatics, 23, i41–i48 17646325 10.1093/bioinformatics/btm229 PMC2516305

[4] Bodenreider O. (2008) Ontologies and data integration in biomedicine: success stories and challenging issues. Data Integr. Life Sci., 5109, 1–4

[5] Spasic I. Ananiadou S. McNaught J.*.* (2005) Text mining and ontologies in biomedicine: making sense of raw text. Brief. Bioinformatics, 6, 239–251 16212772 10.1093/bib/6.3.239

[6] Hirschman L. Gully A. Krallinger M.*.* (2008) Text mining for the biocuration workflow. Database, 2012, bas020 22513129 10.1093/database/bas020 PMC3328793

[7] Blaschke C. Leon E.A. Krallinger M. Valencia A. (2005) Evaluation of BioCreAtIvE assessment of task 2. BMC Bioinformatics, 6, S16 15960828 10.1186/1471-2105-6-S1-S16 PMC1869008

[8] Lu Z. Hirschman L. (2012) Biocuration workflows and text mining: overview of the BioCreative 2012 workshop track II. Database (Oxford), 2012, bas043 23160416 10.1093/database/bas043 PMC3500522

[9] Ruch P. (2006) Automatic assignment of biomedical categories: toward a generic approach. Bioinformatics, 22, 658–664 16287934 10.1093/bioinformatics/bti783

[10] Trieschnigg D. Pezik P. Lee V. (2009) MeSH Up: effective MeSH text classification for improved document retrieval. Bioinformatics, 25, 1412–1418 19376821 10.1093/bioinformatics/btp249 PMC2682526

[11] Couto F. Silva M. Coutinho P. (2005) Finding genomic ontology terms in text using evidence content. BMC Bioinformatics, 6(Suppl. 1), S21 15960834 10.1186/1471-2105-6-S1-S21 PMC1869014

[12] Krallinger M. Padron M. Valencia A. (2005) A sentence sliding window approach to extract protein annotations from biomedical articles. BMC Bioinformatics, 6(Suppl. 1), S19 15960831 10.1186/1471-2105-6-S1-S19 PMC1869011

[13] Gaudan S. Yepes A.J. Lee V. Rebholz-Schuhmann D. (2008) Combining evidence, specificity, and proximity towards the normalization of Gene Ontology terms in text. EURASIP J. Bioinform. Syst. Biol.*,* 2008, 342746. doi: 10.1155/2008/342746 10.1155/2008/342746 PMC3171395 18437221

[14] Winnenburg R. Wächter T. Plake C. (2008) Facts from text: can text mining help to scale-up high-quality manual curation of gene products with ontologies? Brief. Bioinform*.*, 9, 466–478 19060303 10.1093/bib/bbn043

[15] Doms A. Schroeder M. (2005) GoPubMed: exploring PubMed with the Gene Ontology. Nucleic Acids Res., 1, 783–786 10.1093/nar/gki470 PMC1160231 15980585

[16] Van Auken K. Fey P. Berardini T.Z. (2012) Text mining in the biocuration workflow: applications for literature curation at WormBase, dictyBase and TAIR. Database (Oxford), 2012, bas040 23160413 10.1093/database/bas040 PMC3500519

[17] Gobeill J. Pasche E. Teodoro D. (2012) Answering gene ontology terms to proteomics questions by supervised macro reading in Medline. In: Proceedings of NETTAB Conference, Como, Italy, EMBnet.journal, North America*,* Vol. 18

[18] Gobeill J. Pasche E. Vishnyakova D. Ruch P. (2013) Managing the data deluge: data-driven GO category assignment improves while complexity of functional annotation increases. Database (Oxford)*,* 2013, bat041 23842461 10.1093/database/bat041 PMC3706742

[19] Van Auken K. Schaeffer M. McQuilton P. (2013) Corpus construction for the BioCreative IV GO Task. BioCreative IV Proceedings, NCBI, Bethesda, Maryland, USA.

[20] Huang J. Lu J. Ling C. (2003) Comparing naive bayes, decision trees, and SVM with AUC and accuracy. In: Proceedings of the Third IEEE International Conference on Data Mining (ICDM ’03). IEEE Computer Society, Washington, DC

[21] Colas F. Brazdil P. (2006) Comparison of SVM and some older classification algorithms in text classification tasks. Artif. Intell. Theory Pract., 217, 169–178

[22] Manning C.D. Schütze H. (2009) Foundations of Statistical Natural Language Processing. MIT Press, Cambridge, MA

[23] Ounis I. Amati G. Plachouras V. (2006) Terrier: A High Performance and Scalable Information Retrieval Platform. In: Proceedings of ACM SIGIR'06 Workshop on Open Source Information Retrieval, Seattle, Washington, USA.

[24] Eisner R. Poulin B. Szafron D. (2005) Improving protein function prediction using the hierarchical structure of the Gene Ontology. In: Proceedings of 2005 IEEE Symposium on Computational Intelligence in Bioinformatics and Computational Biology, Edmonton, Alberta, Canada.

[25] Guha R. Gobeill J. Ruch P. (2009) GOAssay: from gene ontology to assays identifiers – towards automatic functional annotation of PubChem BioAssays, In: Nature Precedings. http://dx.doi.org/10.1038/npre.2009.3176.1

[26] Anton B. Chang Y. Brown P.*.* (2013) The COMBREX project: design, methodology, and initial results. PLoS Biol., 11, e1001638 24013487 10.1371/journal.pbio.1001638 PMC3754883

